# Cryo-Structured Chitosan Sponges with Controlled Release Properties for Liquid Digestate as Potential Agricultural Fertilizers

**DOI:** 10.3390/gels11110887

**Published:** 2025-11-04

**Authors:** Marinela Victoria Iordanescu, Alin Cristian Vintilă, Grigore Psenovschi, Luiza Capra, Ana-Mihaela Gavrilă, Cristina Emanuela Enascuta, Tanța-Verona Iordache

**Affiliations:** Advanced Polymer Materials and Polymer Recycling Group and Bioresources Group, National Institute for Research & Development in Chemistry and Petrochemistry-ICECHIM, 202 Spl. Independenței, 060021 Bucharest, Romania; marinela.dumitru@icechim.ro (M.V.I.); alin-cristian.vintila@icechim.ro (A.C.V.); grigore.psenovschi@icechim.ro (G.P.);

**Keywords:** chitosan, cryo-structured sponges, controlled release, macronutrients, agriculture

## Abstract

Phosphorus and potassium are two of the most essential macronutrients that often work together to support plant health and productivity. If one of these elements is deficient, it can lead to reduced plant growth, low yields, and poorer crop quality. For this reason, fertilizers contain these nutrients to replenish soils that have been depleted over time. As a sustainable approach, this study proposes new cryo-structures based on chitosan and liquid digestate with controlled release properties for potassium and phosphorus. For this purpose, commercial chitosan (a biopolymer extracted from marine waste) was used along with a liquid digestate, obtained through anaerobic digestion, to develop cryo-structured sponges. The incorporation of liquid digestate in the cryo-structured sponges was confirmed using different characterization techniques (FTIR, TGA/DTG, SEM, and EDX), while the release mechanism for phosphorus and potassium was investigated using ICP-OES spectroscopy.

## 1. Introduction

Agriculture and food security are facing multiple challenges that affect the entire food supply chain worldwide [1]. The greatest concern is providing food for all human beings in the long term, as the global population is rapidly expanding [2]. Due to this pressure, researchers and farmers have focused on solutions for increasing food productivity, e.g., vertical cropping, urban agriculture, or the use of organic fertilizers to protect soil—targeting multiple vegetation stages [3]. Anyhow, the key to success is sustainability [4]. To aid sustainable cropping and the conservation of energy and water, many researchers have suggested avoiding harmful chemicals and promoting local production and resources [5]. Ecological conservation, biodiversity, and agricultural education are integrated approaches for envisioning the future of sustainable agriculture in North America [6].

Of similar importance for sustainable cropping is the crop’s nutrition plan, which is essential to maintain a high yield and quality of the final products [7]. An imbalance between nutrient supply and crop demand can reduce growth, increase the incidence of nutrient disorders, and elevate the risk of crop damage and environmental pollution due to the unnecessary accumulation of nutrients in the soil [8]. Nevertheless, fertilizer application according to best practice can overcome these issues and improve soil productivity at the same time [9,10,11].

In this context, the importance of using organic fertilizers has grown considerably in recent decades [12]. The main benefits of using organic fertilizers include soil enrichment, nutrient release capacity, environmental impact reduction, sustainability, improved plant health, carbon sequestration, and many others [13]. One of the organic fertilizers that has received much attention over the past few years is digestate, a nutrient-rich by-product, obtained after the anaerobic digestion of waste [14].

Anaerobic digestion (AD) is a well-known biological process among waste treatment methods because it saves energy, has social and economic benefits, and reduces greenhouse gas emissions and disagreeable odors [15]. This process can produce several bioproducts, including biomass, biopolymers, bioplastics, biofertilizers, and biolipids, in addition to biogas [16,17,18,19,20]. The study by Garcia-Lopez et al. [21] proved that the direct application of digestate can affect the biogeochemical cycling of nutrients and soil functionality. Therefore, finding a more suitable way to apply the digestate without harming the soil is critical. For instance, several authors have reported the development of smart composite fertilizers, either by coating [22] or by encapsulating [23] the fertilizer in a synthetic or natural polymer matrix, targeting their efficiency, environmental safety, and cost-effectiveness.

Nevertheless, in the context of sustainability, many recent studies have reported the preponderant application of biopolymers for coating or encapsulating fertilizers [23,24,25]. Cellulose, starch, chitosan, lignin, and alginate have been the most used [26,27,28,29,30,31]. The reasons for choosing biopolymers over synthetic polymers refer to biodegradability, nontoxicity, and additional organic matter intake [32,33]. On the other hand, the use of biopolymers for composite manufacturing also leads to materials with low chemical resistance and mechanical features, which require chemical modification or mixing with other synthetic polymers [34], thus, partially losing their native properties, such as biodegradability and nontoxicity. For example, Singh and Dhaliwal [32] prepared a xanthan gum-cl-poly (acrylic acid)/AgNPs hydrogel nanocomposite for the release of KCl, while Sarmah and Karak [33] proposed a superabsorbent hydrogel based on modified starch with poly(acrylic acid) for urea encapsulation. However, there is still hope for developing completely sustainable controlled release systems for fertilizers, as described by Skrzypczak et al. [35], in which a multilayer hydrogel capsule for Cu, Mn, Zn, and NPK release was developed using only eggshells, sodium alginate, carboxymethyl cellulose, and starch.

In the spirit of sustainability, the present study describes the usage of unmodified chitosan for preparing spongy cryo-structured gels as controlled release systems for a liquid digestate, obtained by anaerobic digestion, containing phosphorus (P) and potassium (K). The release of P and K from the spongy cryo-structures is further evaluated in water using ICP-OES.

Cryo-structured gels or cryogels are chemically or physically crosslinked porous materials synthesized via cryogelation techniques [36,37], often combining biopolymers (e.g., chitosan, gelatin, and cellulose) with inorganic or organic fillers [38,39]. These sponges are valued for their high porosity, water retention, mechanical resilience, flexibility, biodegradability, and potential for loading and controlled release, and are being increasingly explored for applications in soil conditioning, biofertilizer delivery, wastewater treatment, and biomedical scaffolding [39,40,41].

For obtaining free-standing cryogels by physical crosslinking, several freeze–thaw cycles are usually required [42]. However, in recent studies on this group [42,43], the addition of sodium bicarbonate has significantly improved both the porosity and the mechanical stability of the gels, which were prepared using a different approach, e.g., single freezing cycle–lyophilization–rehydration. Thus, different from the procedure used for physically crosslinked cryogels [44], in this study, an additional porogen, e.g., sodium bicarbonate, is used to create a spongy appearance, after which, the material is frozen and lyophilized to enhance its mechanical stability [43].

## 2. Results and Discussion

### 2.1. Digestate Preparation and Content Analysis

During the anaerobic co-digestion process (set-up given in Figure S1 in Supplemental Material), the bio-methane flow rate (DM) and total bio-methane volume (VTM) were monitored and are shown in Table 1, pointing out significant differences between the four investigated compositions, noted as E1, E2, E3, and E4. E1 showed a relatively low DM of 22.39 Nml/h on the first day but showed a maximum of 42.52 Nml/h on the second day, followed by a significant decrease on the following days. E2, E3, and E4 presented a burst on the first day of 54.65 Nml/h, 71.12 Nml/h, and 70.99 Nml/h, respectively, after which they decreased on subsequent days. It is important to note that E3 and E4 presented similar values of DM during the four-day interval, except for the second day, when a sharper decrease in DM was recorded for E4.

The values of VTM obtained in each experiment also reveal significant variations in bio-methane production over the four days. E1 and E2 show a small increase on the first day, after which the VTM increases up to 1700–1800 NmL. For E3 and E4 the VTM values on the first day are similar to the ones obtained for E1 and E2 on the fourth day, and reach values over 2100 NmL at the end of the experiment. From the analyzed data, the optimum recipe for obtaining high bio-methane yields, with VTM values over 2440 NmL per day, was E3.

After complete digestion, the resulting liquid and the solid phases were visually and physically analyzed (results given in Table S1 of the Supplemental Material) and their content of P and K was quantified using ICP-OS (Table 2).

The concentrations of phosphorus (P) and potassium (K) recovered from both solid and liquid digestates in samples E1–E4 (Table 2) demonstrate promising levels. Notably, solid digestates consistently yielded high P values (ranging from 1.00% to 1.57%) and K values (1.54% to 1.89%), which are in line with or exceed concentrations reported in comparable studies [45,46]. Liquid digestates also retained measurable nutrient levels, with P ranging from 0.077% to 0.097% and K from 0.34% to 0.38%. These results underscore the efficiency of the anaerobic digestion process used and reinforce the suitability of these recovered nutrients for reuse in controlled release fertilizer formulations [47].

Among the four samples analyzed, E3 and E4 registered notable concentrations of phosphorus and potassium in both solid and liquid digestates. These samples exhibited P levels of 1.37–1.57% and K levels of 1.58–1.89% in the solid phase, making them particularly suitable for evaluating their compatibility with carrier matrices in controlled release fertilizer formulations. Compared with [47], where the percentage of P and K elements in liquid or solid formulation was around 0.20–0.30% (liquid) and 10–30% (solid), it can be deducted that the nutrient profiles from this current study present a greater potential for integration into sustainable agricultural products [48].

### 2.2. Stability of Unloaded Cryo-Structured Chitosan Sponges as a Function of pH

**Figure 1:**
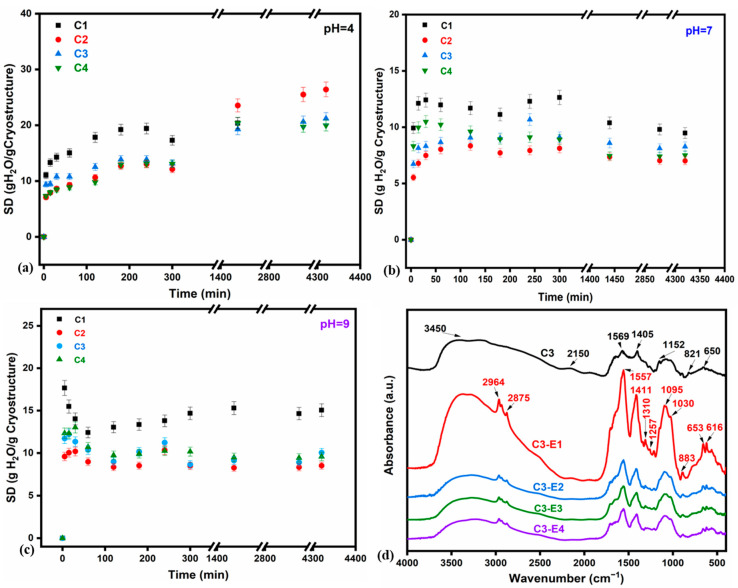
Variation in SDs as a function of time (5% error bars of measured data) for the C1, C2, C3, and C4 cryo-structures, performed at room temperature and different pH values: (**a**) pH 4, (**b**) pH 7, (**c**) pH 9; and comparison of FTIR spectra for samples C3, C3-E1, C3-E2, C3-E3, and C3-E4 (**d**).

The cryo-structures prepared using various concentrations of CC, noted as C1 (0.3 g CC), C2 (0.45 g CC), C3 (0.5 g CC), and C4 (0.5 g CC), showed different behavior during swelling assays. Because the structures were not chemically crosslinked, they tended to disintegrate after 24 h, requiring the timely monitoring of the swelling process. However, our primary interest was to evaluate the SDs at pH 7, the pH of digestate, and ensure that cryo-structures were able to withstand the conditions imposed for digestate adsorption. Thus, the first step in assessing their stability was to evaluate their swelling behavior over time as a function of pH (Figure 1, Table S2—Supplemental Material). The recordings highlight a rather low stability of C1 at all three studied pH values compared with the other three samples, which resisted up to 72 h; C1 disintegrated after nearly 24 h at pH 4 and broke down into smaller pieces at higher pH values. At pH 9, the adsorption was fast for all cryo-structures (with maximum adsorption within the first minutes after contact), followed by a shallow equilibrium as the salt ions are adsorbed into the chitosan structure, blocking further water adsorption. Nevertheless, the SDs recorded at pH = 7 indicate that all four cryo-structures retain large amounts of solution in the first 4 h (240 min), after which they either desorb or lose small pieces of material along the way. It can also be noted that C2 presented the smallest SDs within the analyzed pH interval, while C4, which differs from C3 in its porogen ration, adsorbed higher amounts of water in the first 60 min vs. C3, at pH 7 and 9, after which it followed an unbalanced trend particularly at pH 4 and 9. Thus, from analyzing the SD values within the studied timescale, it can be observed that the C3 cryo-structure had the capacity to adsorb more uniformly relevant amounts of solution (up to 10.67 g H_2_O/g cryo-structure) in 4 h and it was also stable for longer periods of time; the latter is indicated by the slower apparent desorption after 4 h. For this reason, the following studies were performed for C3 alone and C3 loaded with liquid digestate. Overall, the results suggest that near pH 7, the adsorption of water is more homogenous in time and the swelling is slightly lower compared with pH 4 or pH 9, which delays the fragmentation of cryo-structures thus making possible both the loading and the following release of digestate near pH 7.

### 2.3. Structure and Thermal Behavior of Digestate-Loaded Cryo-Structured Chitosan Sponges

The chemical structures of C3 samples loaded with liquid digestates (E1÷E4), namely C3-E1, C3-E2, C3-E3, and C3-E4 (Figure 1d), were expected to be similar as they differ only by the type of liquid digestate. Bands characteristic to chitosan can be identified at 3450 cm^−1^, 1569 cm^−1^, and 1405 cm^−1^, due to O–H from hydroxyl group (moisture), C=O stretching of amide 1, and N-H bending of amide II, respectively [49]. As we move to digestate-based C3 samples, specific bands of macroelements, P and K [50,51], are distinguished: at 1310 and 650 cm^−1^ for K; 1257 cm^−1^, 1152 cm^−1^, and 1030 cm^−1^ corresponding to the stretching vibration of P=O bonds, P–O–C, and P–O; 616 cm^−1^/653 cm^−1^ for O–P–O; and 883 cm^−1^ for calcium carbonate.

In addition to FTIR, thermogravimetric analysis (Figure S2 in Supplemental Material) also indicated that all cryo-structures were loaded with a significant amount of digestate, by exhibiting a supplementary degradation stage in the 90–210 °C temperature range (see Table 3). As can be observed, C3 alone presented only one degradation stage with a maximum degradation temperature of 271.41 °C, while all four loaded cryo-structures presented an intermediary degradation stage, with maximum degradation temperatures between 122 and 131 °C, typically associated with the decomposition of small organic fragments and solvent evaporation [51]. It is also important to note that C3-E3 and C3-E4 exhibited higher thermal stability (with maximum decomposition temperatures shifted to higher temperature values) compared with C3-E1 and C3-E2; this could indicate the formation of more compact structures.

The summarized values of total mass loss, shown in Table 3, also reflect an important residue remaining at 700 °C for all four cryo-structures loaded with digestate (i.e., C3-E1 30.96%, C3-E2 29.30%, C3-E3 27.46%, and C3-E4 27.919%) compared with C3 alone (3% residue), which may be associated with the overall carbon content (C3, as well as organic residues found in the digestate) and inorganic content (macronutrients).

### 2.4. Morphology and Mineral Content Evaluation of Digestate-Loaded Cryo-Structured Sponges

**Figure 2:**
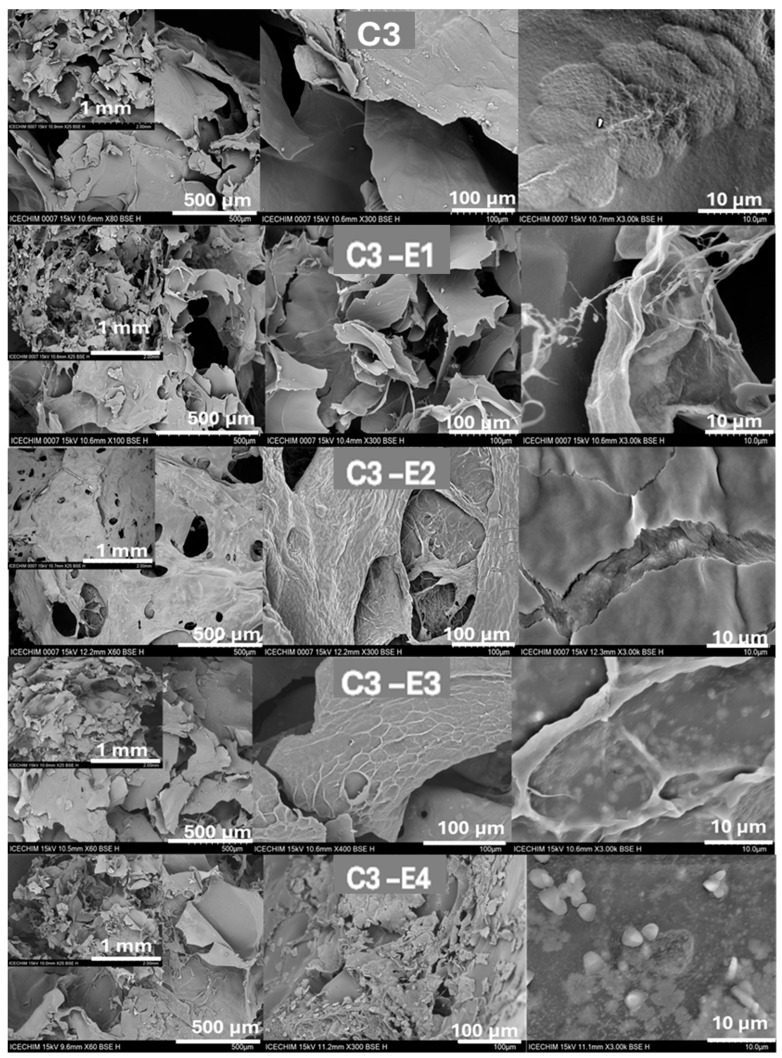
SEM images for lyophilized samples C3, C3-E1, C3-E2, C3-E3, and C3-E4 at 1 mm (medallion), 500 µm, 100 µm, and 10 µm scale.

Complementary to the structural analysis, the morphology analyzed using SEM (Figure 2) revealed the macroporous morphology of C3, C3-E1, C3-E2, C3-E3, and C3-E4 as well as the incorporation of the liquid digestate into the chitosan matrix. Since the main difference between the cryo-structures is the type of incorporated liquid digestate, the morphology of C3 alone and its digestate-loaded homologues is similar. In Figure 2, it can be observed that the layered porous architecture of C3 is mainly preserved after the adsorption process of liquid digestate, indicating that the chitosan matrix was not significantly affected by the adsorption procedure. Nonetheless, the SEM images recorded at 1 mm and 500 µm show that after nutrient adsorption, the morphology of samples becomes more compact, especially for C3-E3, which can prevent burst nutrient release [52]. Although a similar amount of liquid digestate was incorporated into the four samples, only for C3-E3 and C3-E4 can white dots be noticed on the surface of the cryo-structured chitosan matrix, which represent the micronutrients solidified after lyophilization. In the case of C3-E4, some agglomerates are also observed as bigger solid grains.

Sustaining the evidence of digestate incorporation, the EDX spectra (Figure S3, Supplemental material) highlighted the presence of significant amounts of P and K, as well as other elements like Mg, Na, and Ca, on the surface of the cryo-structures (as summarized in Table 4). Compared with the C3 reference, which contained mainly oxygen and carbon, C3-E1 registered 8.4% K and 1.6% P; C3-E2 7.7% K and 2.4% P; C3-E3 11.5% K and 2.5% P; and C3-E4 11.4% K and 4.7% P. By analyzing the content of each element, it can be observed that C3-E4 contained higher amounts of K and P, thus corroborating well with the SEM micrographs. Thus, due to the low amounts of adsorbed microelements, determined by EDX, the C3-E1 and C3-E2 cryo-structures were set aside for the following investigations. As a slight observation, it is worth mentioning that the presence of gold (Au) (Table 4) is attributed to the method used for acquiring the SEM images, as the samples were sputter-coated with a thin layer of gold to enhance conductivity. The detected aluminum (Al) (Table 4) content is likely due to the lyophilization process, during which the samples were covered with aluminum foil to prevent vacuum-induced deformation during extraction.

### 2.5. Adsorption and Controlled Release Studies

#### 2.5.1. Investigation of Liquid Digestate Adsorption by C3 Cryo-Structures

To have better control of the following nutrient release, the adsorption of K and P from the digestate solution by the C3 cryo-structures was monitored by quantifying the amounts of microelements in the solid cryo-structures (as shown in Table 5) after 24 and 48 h adsorption of digestate. The analysis of the solid cryo-structures indicates that both types of C3 cryo-structures contained almost double the amount of P and K after 48 h vs. values registered after 24 h. It is also noteworthy that the adsorption results corroborate well with the TGA of residues after decomposition, in which case around 29% appeared to be carbon and inorganic matter. Hence, we can assume that around 12.56% and 13.78% represent the total of content of P and K, in C3-E3 and C3-E4, respectively.

#### 2.5.2. Controlled Release of P and K from Digestate-Loaded Cryo-Structures

**Figure 3:**
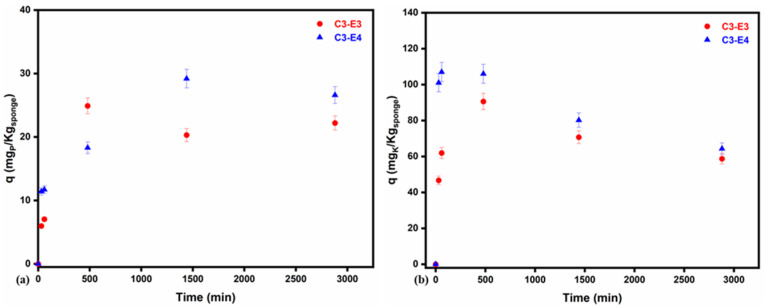
Controlled release of P (**a**) and K (**b**) from C3-E3 and C3-E4 with 5% error bars of measured data.

The controlled release of P and K from C3-E3 and C3-E4 loaded with digestate during 48 h was monitored in demineralized water at pH 7 and analyzed using ICP-OES (Figure 3; also see Table S3, Supplemental material). Figure 3 shows a different release behavior for the two macroelements. For both cryo-structures, a burst release of K was registered in the first 30 min, up to 56.3 × 10^−3^ mg/g and 122.0 × 10^−3^ mg/g, for C3-E3 and C3-E4, respectively. These results are similar to those reported in [53], where green composite materials based on polyhydroxybutyrate, starch, and montmorillonite were used for the controlled release of NPK fertilizers. According to other studies [54], the controlled release capacity for phosphorus (P) and potassium (K) may be comparable, slightly higher, or lower depending on the formulation. However, the type of soil and the specific nutrient requirements of the plants play a crucial role in determining the effectiveness of P and K delivery.

Furthermore, as initially presumed by analyzing its morphology, C3-E3 was able to diminish the burst release of K due to its more compact architecture; the release in the first 60 min was two times lower compared with C3-E4. Nevertheless, the maximum released amount of K for both types of cryo-structures was registered in the first 8 h. On the other side, the release of P was gradual for both cryo-structures, with maximum released amounts after 8 h for C3-E3 (57.1 × 10^−3^ mg/g) and after 24 h for C3-E4 (66.9 × 10^−3^ mg/g).

For evaluating the kinetics of the controlled release capacity for phosphorus (P) and potassium (K), pseudo-first-order, pseudo-second-order, and Elovich kinetic models, given by Equations (2)–(4), respectively [55,56], were used. The graphs were collected in Figures S4a, S5a, and S6a for P, and Figures S4b, S5b, and S6b for K, in the Supplemental Material, while the parameters are summarized in Table 6. For the controlled release of P, the kinetic model suitability ranked as follows: (i) pseudo-first-order model > pseudo-second-order model > Elovich model for the C3-E3 system; (ii) Elovich > pseudo-second-order model > pseudo-first-order model for the C3-E4 system. On the other hand, for K controlled release, the obtained sequence was as follows: (i) pseudo-first-order model > pseudo-second-order model > Elovich model for both systems, C3-E3 and C3-E4. From this sequence, it can be noticed that the C3-E3 system led to similar controlled release mechanisms for both P and K. Meanwhile, the C3-E4 system presented quite different controlled release mechanisms for P and K when fitted to the different kinetic models. Suitability with the pseudo-first-order kinetic model for C3-E3 (K and P) and C3-E4 (P) suggests that the entire process is governed by diffusion, where nutrients are weakly bound to the surface of the adsorbent and released rapidly during the initial phase [57]. Meanwhile, the suitability with the Elovich kinetic model indicates that the C3-E4 system may retain K by chemisorption mechanisms as well [58]. This model is particularly suitable for describing gradual and sustained release from porous or chemically interactive matrices, such as biochar, zeolite, or cryo-structured sponges, as well as in this study [57,58,59].

According to the literature [59], a pseudo-first-order kinetic model also provided the best fit for urea and composite materials containing urea and chitosan. This may indicate that pseudo-first order kinetics are suitable for chitosan-based composite materials. Nevertheless, the Elovich kinetic model was also identified as the best fit for the controlled release of phosphorus (P) in [60], which aligns with the findings of the present study.

Nevertheless, in natural environments, such as water and soil, chitosan degrades into D-glucosamine and N-acetyl-D-glucosamine by enzymatic, chemical, and microbial processes [61]. Hence, taking into consideration both the natural factors and the pH stability, it can be presumed that in natural environments, the fragmentation of cryo-structures occurs faster, which could lead to burst microelement release after each fragmentation step.

## 3. Conclusions

This study focused on combining liquid digestate (obtained through anaerobic digestion) with cryo-structured chitosan sponges to develop potential fertilizer materials. To this end, chitosan and liquid digestate were used to prepare cryo-structured chitosan sponges designed for the controlled release of phosphorus (P) and potassium (K) in aqueous solutions.

The liquid digestate was adsorbed onto the chitosan sponges, after which the controlled release capacity was evaluated to determine whether these materials could serve as viable fertilizers. FTIR and SEM/EDX analyses provided evidence of digestate adsorption into the chitosan matrix, revealing changes in the surface morphology and porosity of the resulting cryo-structured sponges. Furthermore, a swelling study conducted at three different pH values indicated that the optimal pH range for the controlled release of P and K lies between pH 4 and pH 7. Consequently, performing the release trials at pH 7.0 resulted in an improved nutrient release performance, comparable to findings reported in similar studies [62,63]. The C3-E4 cryo-structured sponge demonstrated high nutrient release capacities, reaching up to 122 mg K/g and 66.9 mg P/g. Based on regression analysis, the pseudo-first-order kinetic model provided the best fit for the release of P from both analyzed cryo-structures, while Elovich was quite suggestive for K release from C3-E4. Therefore, the proposed cryo-structured chitosan sponges show promise as controlled release fertilizer materials for P and K in aqueous environments, and potentially for future application in agriculture.

It is also important to mention that the storage of liquid digestate in the sponges is limited by the stability of the cryo-structures at pH 7, e.g., up to 48 h. However, if lyophilized and kept in dried and light-protected spaces that prevent chitosan biodegradation, the sponges may store the micronutrients until use for longer periods of time, up to 6 months.

As a final remark, the materials developed in this study exhibit promising environmental benefits due to their biodegradability and low production cost. Their sponge-like structure and nutrient retention capabilities make them suitable candidates for sustainable soil amendment applications. However, further research is needed. For this reason, future work will address field trials and soil incubation studies to validate the practical performance and agronomic relevance of the developed materials as well as greenhouse experiments to evaluate the agronomic performance, nutrient uptake efficiency, and long-term effects on soil health and crop development.

## 4. Materials and Methods

### 4.1. Materials

To obtain the desired cryo-structures, commercial chitosan (CC, ≥75% deacetylation degree, Mn = 2.056 × 105 g/mol, supplied by Sigma-Aldrich, Louis, MO, USA) was used as received. Standard solutions of pH 4, pH 7, and pH 9 (supplied by Metrohm Ltd., Herisau, Switzerland) were used for the determination of the swelling degree. For the dissolution of chitosan, a mixture of glacial acetic acid (99%, Sigma-Aldrich) and distilled water was used. Ammonium bicarbonate (NH_4_HCO_3_, 99.5%, Sigma-Aldrich) was used as a foaming agent. The liquid digestate was prepared by anaerobic digestion process in our laboratories (National Research and Development Institute for Chemistry and Petrochemistry), similarly to the previous work of [64].

### 4.2. Characterization Techniques

#### 4.2.1. Chemical Composition by Fourier Transform Infrared (FTIR) Spectroscopy

The FTIR spectra were recorded using a Thermoscientific Summit Pro spectrophotometer (Thermo Fisher Scientific, Waltham, MA, USA), performing 16 scans for each sample, at a resolution of 4 cm^−1^, in the spectral range 4000–400 cm^−1^. The samples were analyzed as potassium bromide pellets.

##### Scanning Electron Microscopy (SEM)

SEM images were recorded using a Quanta Inspect F Scanning Electron Microscope (Thermo Fisher Scientific manufacturer, Waltham, MA, USA) equipped with an emission gun, a 1.2 resolution field (EGF), and an X-ray energy dispersion spectrometer (EDS). SEM images were taken for all the cryo-structures to confirm the encapsulation of the liquid digestate. The samples were placed on a carbon strip, which was further placed on a copper grid. The samples were coated for 30 s with a thin layer of gold.

##### Thermogravimetric Analysis (TGA)

TGA and DTG thermogravimetric investigations of the obtained cryo-structures were carried out using the TA Q500 instrument (TA Instruments, New Castle, DE, USA). Each sample was analyzed in the temperature range 25–750 °C at a heating rate of 10 °C/min in a nitrogen atmosphere.

##### Inductively Coupled Plasma Optical Emission Spectroscopy (ICP-OES)

The Optima 2100 DV ICP-OES System (Perkin Elmer, Waltham, MA, USA) was used for the determination of P and K content, with a dual view optical system—an axial and radial view of the plasma in a single working sequence—which works with an independent transistorized radio frequency generator with a frequency of 40 MHz. The nebulization system is equipped with a PEEK Mira Mistâ nebulizer coupled with a Baffled Cyclonic spray chamber. The spectrometer consists of an optical module comprising an Echelle monochromator with a two-dimensional CCD (charged-coupled device) detector, with the spectral range being 165–800 nm.

All reagents used in the achievement of the experiments for the quantitative determination of the elements listed above were of analytical purity. Centipur Standard solutions, at concentrations of 1000 mg/L and 100 mg/L, respectively, from Merk (Darmstadt, Karlsruhe, Germany) were used. Ultrapure water produced by Milli-Q, Integral System (Merk) with a resistivity of 18.2 MΩ/cm was used for the preparation of the working solutions and samples.

The purging gas for ICP-OES was Argon 5.0 of purity 99.999% (Messer Romania Gaz SRL, Bucharest, Romania). The selected and optimized operating parameters for P and K measurement by ICP-OES were the Radial module with attenuation, at a generator power of 1.25 kW, at a flow rate of argon of 0.75 mL/min, a plasma flow rate of 15 mL/min, an auxiliary argon flow rate of 1.5 mL/min, and peristaltic pump flow rate 1.5 mL/min, with a total analysis time approx. 110 sec/sequence.

### 4.3. Preparation of Digestate

The digestate was prepared using the same amounts of chicken manure, tomatoes, whole potatoes, and dried microalgal biomass (used as a photocatalyst to accelerate the anaerobic digestion process and obtain better biomass degradation) while varying the amount of sawdust and water. In this respect, four samples with different substrate compositions for anaerobic co-digestion were prepared (compositions given in Table 7). Experiments on the anaerobic co-digestion process were carried out using the Gas Endeavour equipment (Figure S1). The operating conditions of the equipment during the anaerobic digestion process were as follows: temperature—37 °C (mesophilic anaerobic digestion conditions), stirring speed—100 rpm (in cycles of 20 min on, 5 min off), and the duration of the process was 4 days. After digestion, the residual biomass was subjected to a centrifugation separation step. The two resulting phases, solid and liquid, were analyzed to determine the macroelement content. Experiment 1 (noted as E1, in Table 7) was used as a reference recipe for optimizing the composition that yields a higher volume and a higher flow rate of bio-methane as well as a digestate rich in P and K.

### 4.4. Preparation of Cryo-Structured Sponges

Several cryo-structures were prepared using various concentrations of commercial chitosan (CC) as follows: C1 (0.3 g), C2 (0.45 g), C3 (0.5 g), and C4 (0.5 g). For each trial, a glass beacon of 25 mL was used to solubilize CC in 12 mL of acetic acid solution (1%) at a gravimetric ratio between CC/solvent of 1:40 (C1), 1:27 (C2), 1:24 (C3), and 1:24 (C4) under magnetic stirring (room temperature, 3 h). When a homogeneous distribution is reached, the foaming agent (NH_4_HCO_3_) is added in a mass ratio CC:NH_4_HCO_3_ of 1:1.33 (C1), 1:2 (C2), 1:1.2 (C3), and 1:2 (C4), after which the mixture is homogenized with a spatula, until the foaming of the chitosan solution is complete. Freeze-drying of the samples was carried out in two steps: (i) primary freezing of the bulk sample (120 h, −20 °C); (ii) lyophilization (72 h, −55 °C), using frozen samples, freshly cut to approx. 1 cm^3^ (photographs of samples given in Figure S7—Supplemental Material). The cryo-structures with liquid digestate content were prepared by the adsorption of liquid digestate (E1, E2, E3, E4) at different gravimetric ratios between CC/digestate of 1:222 (E1), 1:230 (E2), 1:192 (E3), and 1:172 (E4), at room temperature for 48 h (photographs of samples given in Figure S8—Supplemental Material). The pH of the liquid digestate was measured with a CONSORT C931 multimeter and corrected to pH 7 by the addition of 1 M NaOH and 1 M H_2_SO_4_, prior to adsorption.

### 4.5. Determination of P and K Content from Digestate

For determining the content of P and K in the solid and liquid phases, an amount of 0.6 g of solid or 2 g of liquid was weighed and mineralized with HCl (37%):HNO_3_ (65%) = 21:7, under reflux for 2 h, followed by the addition of ultrapure water (quantitatively added to a 100 mL glass flask). The analytical determination of the elements was performed by ICP-OES against calibration curves, obtained using standards obtained by dilution from the Certipur standard solution of 1000 mg/L and 100 mg/L. For P, the calibration curve was determined at λ = 213.617 nm, in the concentration range 0.5–10.0 mg/L (r = 0.9999), while for K, the calibration was performed at λ = 766.490 nm in the concentration range 1.0–8.0 mg/L (r = 0.9997). In this respect, the linearity of the calibration curve was evaluated using the regression function. The acceptance criterion for linearity is the correlation coefficient r, with values of r ≥ 0.997. The elemental concentrations of the samples analyzed are expressed as mass% relative to the total sample mass. P and K determined in the liquid and solid phases of the digestate were compared to the initial content found in the liquid and solid phases of the raw mixture. The same procedures were applied for determining the content of P and K in the solid cryo-structures with the difference that 0.4 g of cryo-structured material was used.

### 4.6. Determination of Swelling Degree (SD)

The swelling capacity of the cryo-structures was studied for 3–4 h at various pH values. The samples were soaked in 10 mL of pH standard solution (4, 7, and 9), in Falcon tubes, with a capacity of 50 mL, with the stirring being ensured by a Multi-Therm shaker device (Cool-Heat-Shake, Benchmark Scientific Inc., Sayreville, NJ, USA) Benchmark (200 rpm, 22 °C). SDs were determined at different time intervals and were assessed according to Equation (1):

(1) $$SD=\frac{m_{swelled}-m_{dried}}{m_{dried}}$$

### 4.7. Controlled Release Studies of P and K from Digestate-Loaded Cryo-Structures

The cryo-structured chitosan sponges were evaluated for their ability to release phosphorus (P) and potassium (K) in a controlled manner in aqueous solution. Experiments consisted of placing 0.03 g of cryo-structured material loaded with liquid digestate in dialysis bags and immersing them in a volume of 10 mL of demineralized water under constant stirring (220 rpm at 20 °C). At 5 different time intervals (30 min, 60 min, 480 min, 1440 min, and 2880 min), 3 mL of supernatant was collected and replaced with fresh demineralized water. The supernatants were analyzed by ICP-OES to quantify the K and P content, according to the methodology described previously.

The kinetics of the controlled release were fitted using a pseudo-first-order model [65], a pseudo-second-order model [66], and the Elovich non-linear model according to Equations (2)–(4), respectively.

(2) $$q_{t}=q_{e}\left(1-\right.\left.e^{-k_{1}t}\right)\;\;\;\;$$

where *q_t_* is the nutrient amount released at time *t*, *q_e_* is the equilibrium nutrient released, *k*_1_ is the release rate constant, and *t* is the time.

(3) $$q_{t}=\frac{q_{e}^{2}k_{2}t}{1+q_{e}k_{2}t\;}$$

where *q_t_* is the nutrient amount released at time *t*, *q_e_* is the equilibrium nutrient released, *k*_2_ is the release rate constant, and *t* is the time.

(4) $$q_{t}=\frac{1}{\beta }\ln\left(1+\alpha \right.\left.\beta t\right)$$

where *q_t_* is the nutrient amount released at time *t*, and *α* (a) and *β* (b) are Elovich parameters.

## Figures and Tables

**Table 1:** Values for bio-methane flow rate (DM) and total volume (VTM) obtained during the anaerobic digestion process.

Exp./Parameter/Day	E1		E2		E3		E4	
	DM (NmL/h)	DM (NmL/h)	DM (NmL/h)	VTM (NmL)	DM (NmL/h)	VTM (NmL)	DM (NmL/h)	VTM (NmL)
1	22.39	54.65	54.65	1311.70	71.12	1707.00	70.99	1703.00
2	42.52	17.97	17.97	1743.00	28.75	2397.10	16.19	2092.50
3	7.00	1.60	1.60	1781.40	1.916	2443.10	1.51	2128.80
4	0.025	0.004	0.004	1781.50	0.025	2443.70	0.03	2129.60

**Table 2:** P and K content from the liquid and solid phases resulting from anaerobic digestion, determined by ICP-OES.

Exp.	Solid Digestate		Liquid Digestate		Initial Mixture			
					Solid		Liquid	
	P (%)	K (%)	P (%)	K (%)	P (%)	K (%)	P (%)	K (%)
E1	1.00	1.54	0.077	0.34	0.90	1.53	0.022	0.26
E2	1.13	1.62	0.089	0.38	1.75	1.54	0.035	0.28
E3	1.37	1.89	0.081	0.36	1.27	1.76	0.030	0.31
E4	1.57	1.58	0.097	0.37	1.35	1.70	0.039	0.29

**Table 3:** Maximum decomposition temperatures and total mass losses evaluated by TGA.

Sample	T_1Max_ °C (Water)	T_2Max_ °C (1st Degradation Stage)	T_3Max_ °C (2nd Degradation Stage)	Total Mass Loss %
C3-E1	63.75	-	271.41	97.00
C3-E2	48.27	122.12	260.17	69.04
C3-E3	46.90	125.36	255.57	70.70
C3-E4	47.25	130.35	267.70	72.54

**Table 4:** EDX results of cryo-structured sponges compared with the C3 reference.

	Element Weight (%)				
Sample/ Element	C3	C3-E1	C3-E2	C3-E3	C3-E4
C	47.1	38.0	35.9	36.5	37.6
O	31.7	26.4	24.0	22.9	26.1
Au	21.2	17.2	21.7	15.9	10.9
P	-	8.4	7.7	11.5	11.4
K	-	1.6	2.4	2.5	4.7

**Table 5:** Adsorbed P and K determined by analyzing the solid C3 cryo-structures after adsorption.

Sample	Time (h)	P_2_O_5_ %(wt.)	K_2_O %(wt.)
C3-E3	24	3.49	2.57
C3-E3	48	6.97	5.59
C3-E4	24	3.27	3.75
C3-E4	48	6.26	7.52

**Table 6:** Parameters for the three investigated kinetic models for P and K release in water.

**Pseudo-first-order kinetic model (Equation (1))**						
	**P**			**K**		
Sample	*K(b)* (g mg^−1^ min^−1^)	*q*_e_(a) (mg/g)	*R* ^2^	*K(b)* (g mg^−1^ min^−1^)	*q*_e_(a) (mg/g)	*R* ^2^
C3-E3	0.008	22.49	0.968	0.03	73.19	0.888
C3-E4	0.013	24.65	0.866	6.90	91.70	0.833
**Pseudo-second-order kinetic model (Equation (2))**						
	**P**			**K**		
Sample	*K*_2_ (g mg^−1^ min^−1^)	*q*_e_ (mg/g)	*R* ^2^	*K*_2_ (g mg^−1^ min^−1^)	*q*_e_ (mg/g)	*R* ^2^
C3-E3	4.3 × 10^−4^	24	0.935	9.43 × 10^−4^	74.05	0.872
C3-E4	5.78 × 10^−4^	27	0.913	1	91.71	0.832
**Elovich non-linear kinetic model (Equation (3))**						
	**P**			**K**		
Sample	*α(a)* (mg g^−1^ min^−1^)	*β(b)*	*R* ^2^	*α(a)* (mg g^−1^ min^−1^)	*β(b)*	*R* ^2^
C3-E3	0.640	0.247	0.867	2.320	0.29	0.810
C3-E4	1.469	0.246	0.954	2.061	1	0.802

**Table 7:** Composition of samples used in the anaerobic digestion process.

Exp.	Potatoes (g)/Tomato (g)/Chicken Manure (g)	Sawdust (g)	Microalgae (g)	Liquid Digestate/Inoculum (g)	Water (g)
E1	48/37/28	30.0	0	40	217.0
E2	48/37/28	23.0	5	40	219.0
E3	48/37/28	15.5	10	40	221.5
E4	48/37/28	8.0	15	40	224.0

## Data Availability

The original contributions presented in this study are included in the article/Supplementary Material. Further inquiries can be directed to the corresponding authors.

## Supplementary Materials

The following supporting information can be downloaded at: https://www.mdpi.com/article/10.3390/gels11110887/s1, Figure S1: Gas Endeavour equipment for the anaerobic digestion process; Figure S2. TGA (a) and DTG (b) for C3 and C3-E1, C3-E2, C3-E3, and C3-E4; Figure S3. EDX spectra of cryo-structured sponges compared with the C3 reference; Figure S4. Non-linear regression using the pseudo-first-order kinetic model applied to controlled release data of P (a) and K (b); Figure S5. Non-linear regression using the pseudo-second-order kinetic model applied to controlled release data of P (a) and K (b); Figure S6. Non-linear regression using the Elovich kinetic model applied to controlled release data of P (a) and K (b); Figure S7. Cryo-structured chitosan sponges after lyophilization; Figure S8. Composite cryo-structured sponges in contact with liquid digestate; Table S1. Physical properties of the liquid and solid fractions resulting from anaerobic digestion; Table S2. Swelling degrees of the cryo-structure series performed at different pH values (4, 7, and 9); Table S3. Released P and K at different time intervals from C3-E3 and C3-E4.
