# Supplementary material for: Cryo-Structured Chitosan Sponges with Controlled Release Properties for Liquid Digestate as Potential Agricultural Fertilizers
Source: Gels. 2025 Nov 4;11(11):887. doi: 10.3390/gels11110887 (PMC12652407; doi:10.3390/gels11110887)
Supplement: Supplementary file 1 [file gels-11-00887-s001.zip › gels-3932618-supplementary.pdf]

Supplemental material

for

Cryo-structured chitosan sponges with controlled release properties for liquid digestate as potential agricultural fertilizers

Experimental

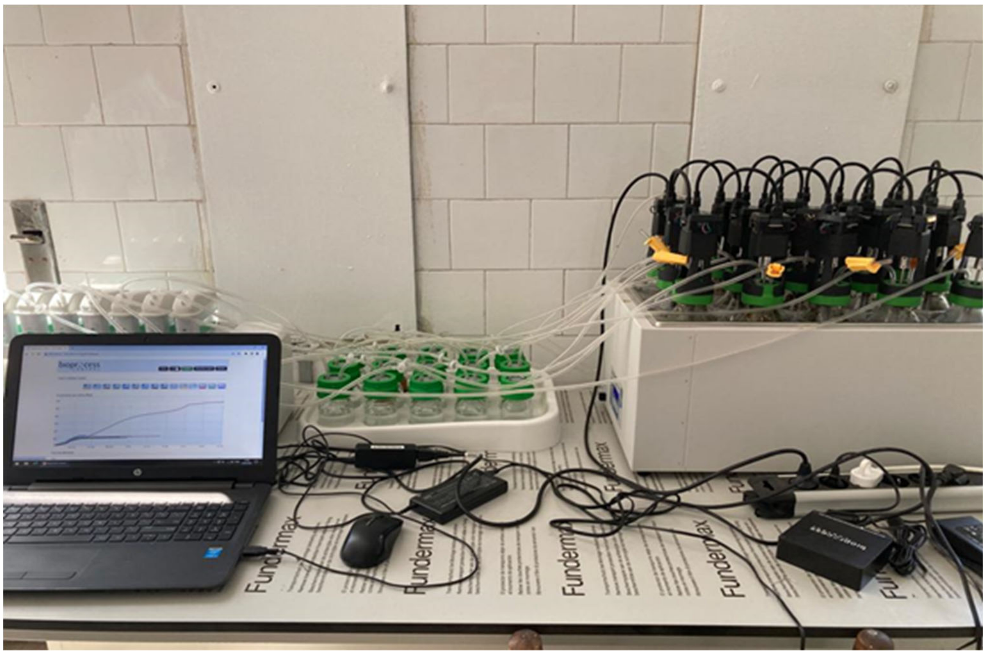

Figure S1. Gas Endeavour equipment for the anaerobic digestion process.

Results & Discussion

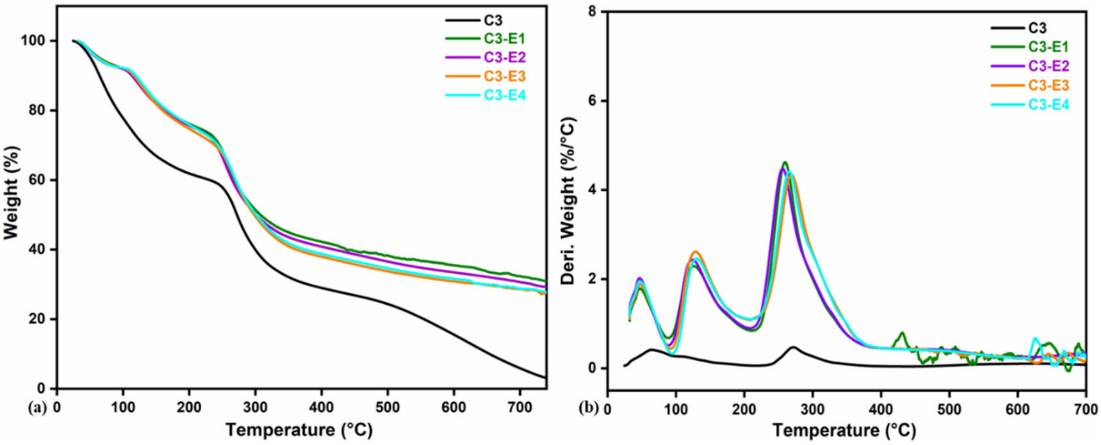

Figure S2. TGA (a) and DTG (b) for C3 and C3-E1, C3-E2, C3-E3 and C3-E4.

Table S1. Physical properties of the liquid and solid fractions resulted from anaerobic digestion.

Exp	Solid fraction of digestate	Liquid fraction of digestate	Initial mixture	
			Solid	Liquid
E1	powder mixture with straw, brown color, 7 g	brown cloudy liquid, 13 mL	powder mixture with straw, brown color, 7g	brown cloudy liquid, 13 mL
E2	powder mixture with straw, brown color, 7 g	brown cloudy liquid, 13 mL	powder mixture with straw, kaki color, 7 g	brown cloudy liquid, 14 mL
E3	powder mixture with straw, kaki color, 7.5 g	brown cloudy liquid, 14 mL	powder mixture with straw, kaki color, 7 g	brown cloudy liquid, 12 mL
E4	powder mixture with straw, kaki color, 7 g	brown cloudy liquid, 13.5 mL	powder mixture with straw, kaki color, 7 g	brown cloudy liquid, 10 mL

Table S2. Swelling degrees of the cryo-structures performed at different values of pH (4, 7 and 9).

Time (min)	*SD* (g H_2O/ g Cryostructure) pH=4				*SD*(gH_2O/g Cryostructure) pH=7				*SD* (g H_2O/ g Cryostructure) pH=9			
	C1	C2	C3	C4	C1	C2	C3	C4	C1	C2	C3	C4
5	11.02	7.09	9.37	7.29	9.91	5.54	6.73	8.32	17.66	9.61	11.72	12.34
15	13.31	7.91	9.49	7.93	12.11	6.79	8.14	9.98	15.47	10.05	12.20	12.34
30	14.26	8.64	10.79	8.45	12.41	7.49	8.32	10.49	14.01	10.20	11.34	13.01
60	15.03	9.30	10.79	8.86	11.97	8.04	8.66	10.23	12.41	9.00	10.37	10.70
120	17.83	10.63	12.52	9.81	11.68	8.35	9.06	9.62	13.04	8.37	9.01	9.74
180	19.21	12.71	13.86	12.95	11.12	7.72	9.03	8.93	13.35	8.54	10.15	9.92
240	19.41	13.05	13.86	13.12	12.28	7.93	10.67	9.10	13.79	10.34	11.23	10.25
300	17.29	12.12	13.16	13.07	12.63	8.12	9.11	8.92	14.67	8.51	8.67	10.17
1440	20.36	23.55	19.27	20.39	10.38	7.36	8.59	7.45	15.29	8.29	9.15	9.50
2880	-	25.50	20.63	19.72	9.79	7.03	8.12	7.41	14.64	8.36	8.92	9.45
4320	-	26.42	21.20	19.97	9.48	7.02	8.27	7.51	15.04	8.54	10.05	9.60

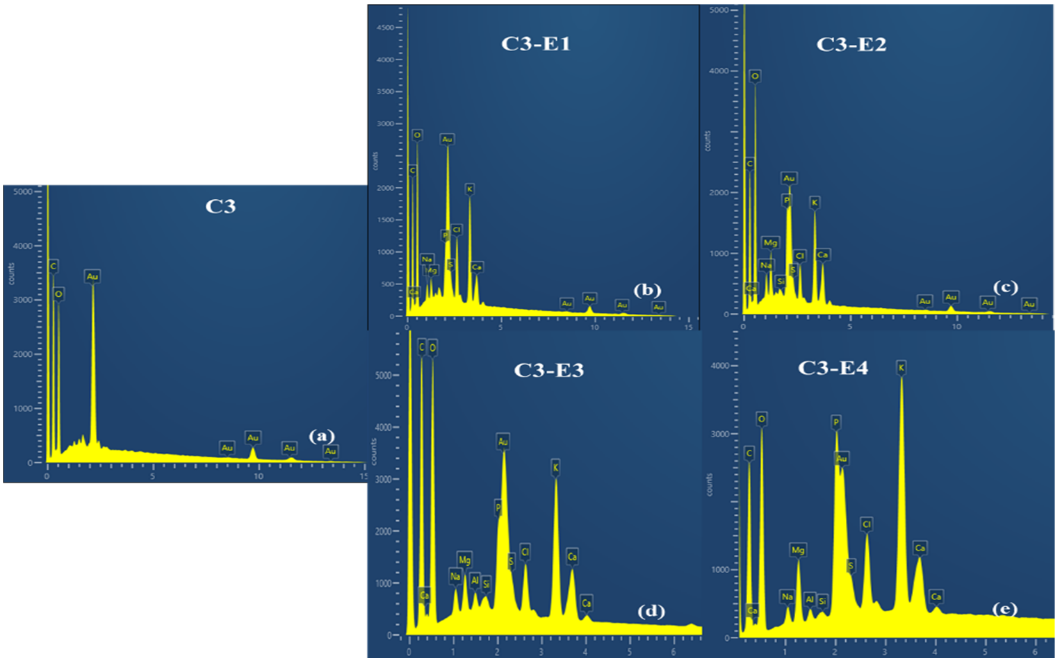

Figure S3. EDX spectra of cryo-structures sponges compared with the C3 reference.

Table S3. Released P and K at different times intervals from C3-E3 and C3-E4.

**Sample**	**Time (min)**	**Elements**	
		**P_2O_5**	**K_2O**
		mg/g (^10^{-3})	mg/g (^10^{-3})
C3-E3	30	13.7	56.3
C3-E4		26.1	122
C3-E3	60	16.1	74.6
C3-E4		26.8	129
C3-E3	480	57.1	109
C3-E4		41.9	128
C3-E3	1440	46.5	85.2
C3-E4		66.9	96.6
C3-E3	2880	50.9	70.7
C3-E4		61.0	77.5

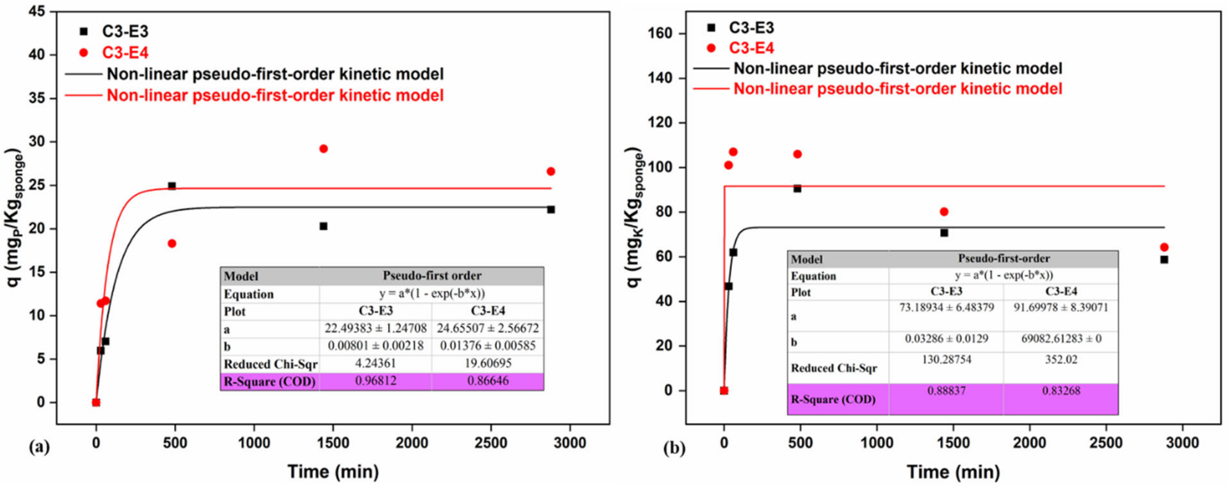

Figure S4. Non-linear regression using the pseudo-first-order kinetic model applied to controlled release data of P **(a)** and K **(b).**

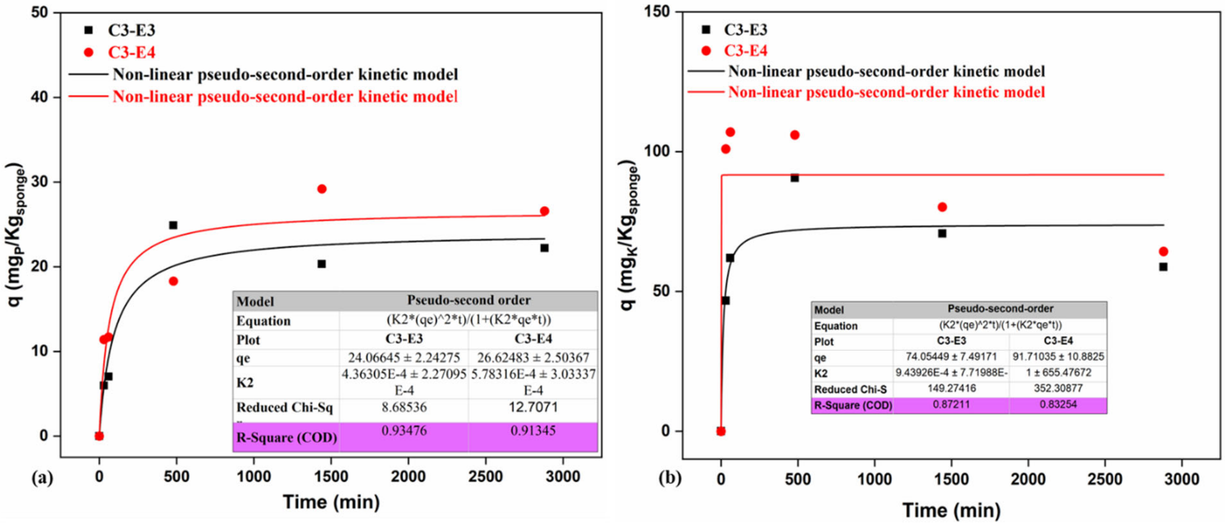

Figure S5. Non-linear regression using the pseudo-second-order kinetic model applied to controlled release data of P **(a)** and K **(b).**

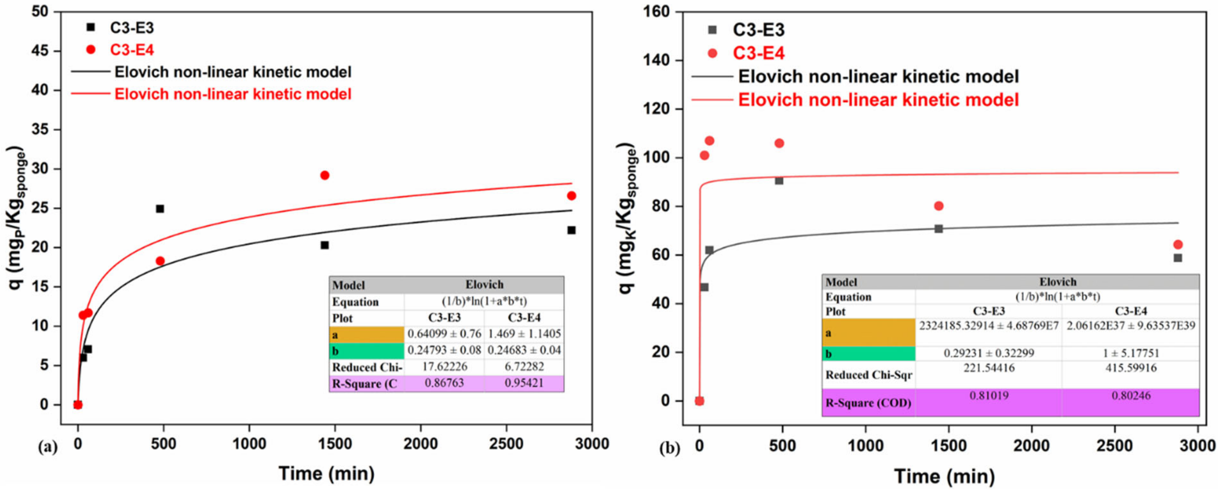

Figure S6. Non-linear regression using the Elovich kinetic model applied to controlled release data of P **(a)** and K **(b).**

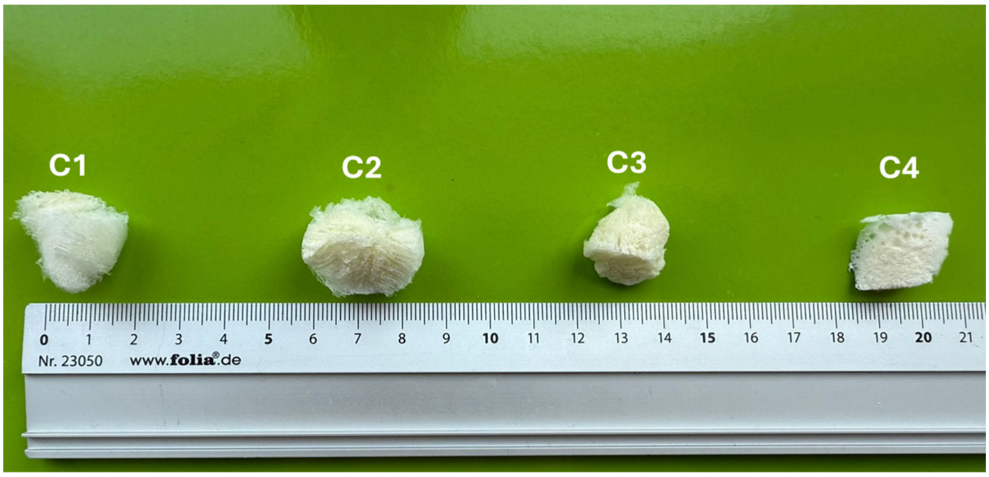

Figure S7. Cryo-structured chitosan sponges after lyophilization.

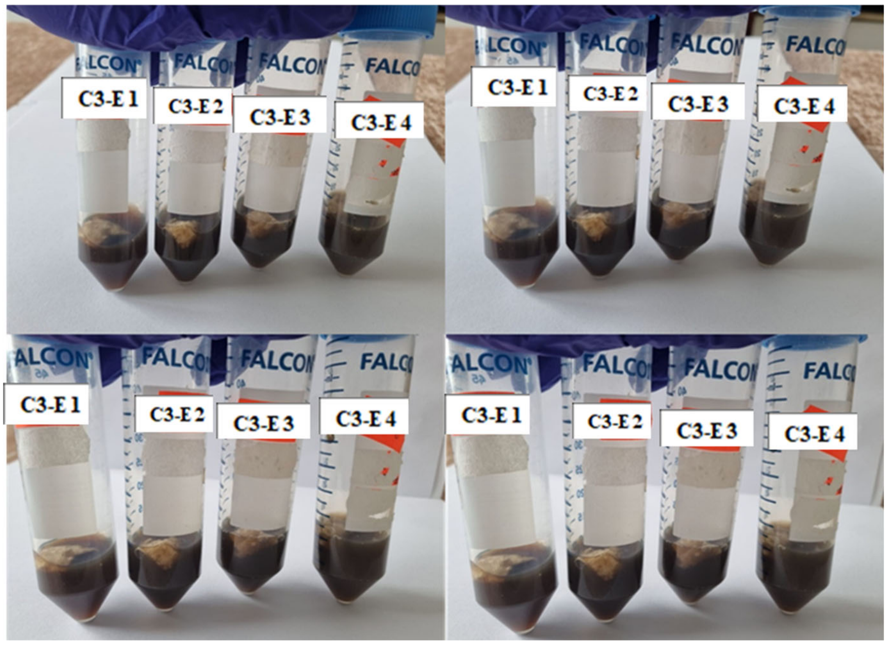

Figure S8. Composite cryo-structures sponges during loading with liquid digestate.
